# Effect of co-inoculation with arbuscular mycorrhizal fungi and phosphate solubilizing fungi on nutrient uptake and photosynthesis of beach palm under salt stress environment

**DOI:** 10.1038/s41598-021-84284-9

**Published:** 2021-03-11

**Authors:** Xue-Ming Zai, Jun-Jun Fan, Zhen-Ping Hao, Xing-Man Liu, Wang-Xiang Zhang

**Affiliations:** 1 Horticulture Department, Jinling Institute of Technology, Nanjing, 210038 People’s Republic of China; 2 Lianyungang Academy of Agricultural Sciences, Lianyungang, 222000 People’s Republic of China; 3 College of Forestry, Nanjing Forestry University, Nanjing, 210037 People’s Republic of China

**Keywords:** Plant physiology, Agroecology

## Abstract

Beach plum (*Prunus maritima*) is an ornamental plant, famous for its strong salt and drought stress tolerance. However, the poor growth rate of transplanted seedlings has seriously restricted its application in salinized soil. This study investigated the effects of inoculation with arbuscular mycorrhizal fungus (AMF), *Funneliformis mosseae*, and phosphate-solubilizing fungus (PSF), *Apophysomyces spartima*, on the growth, nutrient (N, P, and K) uptake, and photosynthesis of beach plum under saline (170 mM NaCl) and non-saline (0 mM NaCl) conditions. We aimed to find measures to increase the growth rate of beach plum in saline-alkali land and to understand the reasons for this increase. The results showed that salinization adversely affected colonization by AMF but positively increased PSF populations (increased by 33.9–93.3% over non-NaCl treatment). The dual application of AMF and PSF mitigated the effects of salt stress on all growth parameters and nutrient uptake, significantly for roots (dry weight and P and N contents increased by 91.0%, 68.9%, and 40%, respectively, over non-NaCl treatment). Salinization caused significant reductions in net photosynthetic rate (*P*_n_), stomatal conductance (*G*_*s*_), transpiration rate (*E*), and intercellular CO_2_ concentration (*C*_i_) value, while inoculation with AMF and PSF inoculations significantly abated such reductions. The maximum efficiency of photosystem II (PSII) (F_v_/F_m_), the photochemical quenching coefficient (qP), and the nonphotochemical quenching (NPQ) values were affected little by inoculation with AMF, PSF, or both under non-NaCl treatments. However, plants inoculated with AMF and/or PSF had higher F_v_/F_m_, qP, and Ф_PSII_ values (increased by 72.5–188.1%) than the control under NaCl treatment, but not a higher NPQ value. We concluded that inoculation with AMF or PSF increased nutrient uptake and improved the gas-exchange and Chl fluorescence parameters of beach plum under salt stress environment. These effects could be strengthened by the combination of AMF and PSF, especially for nutrient uptake, root growth, and *P*_n_, thereby alleviating the deleterious effects of NaCl stress on beach plum growth.

## Introduction

Soil salinization is a global issue that degrades agricultural land and leads to reduced agricultural yields. It has been estimated that saline soils account for about 8% of the Earth’s surface and are increasing globally^1^. In these areas, planting crops or fruit trees adapted to alkali-saline soil has promising prospects. Beach plum (*Prunus maritima*) is a multi-stemmed and deciduous shrub with an extra-long flowering period (more than three months) and rich, edible fruit^2^. Beach plum has an extremely strong ability to tolerate salt and drought stress and grows on the eastern coast of the United States. In 2001, it was first introduced to China by Nanjing University. Although the survival rate is high for beach plum in salinized soil, the poor growth rate of transplanted seedlings had seriously restricted its application in salinized soil in China^3^.

It is well known that the uptake of mineral nutrients by underground roots and the efficiency of photosynthesis of aboveground leaves are "source" forces for plant growth. Higher concentrations of ions (Na^+^, Cl^−^, SO_4_^2−^) in saline soils accumulate in plant cells and inhibit nutrient (e.g., nitrogen, phosphorus, and potassium) uptake^4^ and photosynthesis^5^. In fact, salinity was found to inhibit specific enzymes involved in the synthesis of photosynthetic pigments, resulting in the decrease of chlorophyll content^6^. Salt stress can also cause significant disturbance to chlorophyll fluorescence. The change of rapid chlorophyll fluorescence can reflect the change of photosynthetic electron transfer and the function and stability of photosystem II (PSII)^7^. It is widely regarded as an important parameter for diagnosing the operation of the photosynthetic apparatus in plants and analyzing the mechanism of plant response to stress^8^.

The introduction of arbuscular mycorrhizal fungus (AMF), *Funneliformis mosseae* (formerly known as *Glomus mosseae*), to sites with saline soils may improve the salt tolerance and growth of plants^9^. Colonization of plant roots and soils by AMF could promote plant nutrient acquisition by increasing available phosphorus (P), hydrolyzable nitrogen (N), organic matter content, and several enzyme activities in soil^10,11^. The application of mycorrhizal inoculants could also improve the photosynthesis of plants by increasing Chl content^5^, transpiration rate (*E*), and stomatal conductance (*G*_*s*_) and reducing Na^+^ and Cl^−^ uptake^5,12–14^ in host plants under salt stress. Inoculation of plants with phosphate-solubilizing fungus (PSF), *Apophysomyces spartima*, is also applied for saline soil restoration because it can increase P availability in soils fertilized with rock phosphates^15^. The released P cannot be transferred to the roots by the PSF but may be taken up by the external mycelium of the AMF^16^. Combined AMF and PSF inoculation could also alleviate the effects of salt stress on plant growth by enabling greater nutrient absorption, higher ionic accumulation in different root tissues, and maintenance of lower root Na^+^/K^+^ when salinity is within acceptable limits^17^.

Therefore, this study investigated the following questions. ① Can AMF and/or PSF lessen saline-induced interference of growth, nutrient absorption, and photosynthesis parameters in beach plum? ② How does AM fungi inoculation impart saline tolerance to beach plum?

## Results

### AMF colonization, PSF populations, and plant biomass of beach plum under salt stress

Salinity had a significant adverse effect (*p* < 0.01) on the AMF colonization of beach plum and had a promoting effect on PSF populations (increased by 33.9–93.3% compared to non-NaCl treatment) (Table 1). Mycorrhizal colonization increased 42% and 45% under non-NaCl and NaCl treatments, respectively, in the dually inoculated plants compared to inoculation with AMF alone. PSF populations in soil co-inoculated with both AMF and PSF were 1.67 × 10^4^ and 3.56 × 10^4^ larger in the non-NaCl and NaCl treatments, respectively, than in soil inoculated with PSF alone.

**Table 1:** Effects of *F. mosseae* and/or *A. spartima* on arbuscular mycorrhizal fungi (AMF) colonization, phosphate-solubilizing fungus (PSF) populations, and growth parameters of beach plum under saline and non-saline conditions.

NaCl treatment (mM)	AMF treatment	PSF treatment	AMF colonization (%)	PSF populations (CFU × 10^4^·g^−1^ dry soil)	Shoot dry weight (g·plant^−1^)	Root dry weight (g·plant^−1^)	Root/shoot dry weight ratio
0	− AMF	− PSF	0^d^	0^d^	4.7 ± 0.5^cd^	0.56 ± 0.02^e^	0.12 ± 0.01^e^
	+ AMF	− PSF	48.7 ± 9.9^b^	0^d^	5.6 ± 0.4^b^	0.73 ± 0.01^c^	0.13 ± 0.03^de^
	− AMF	+ PSF	0^d^	0.56 ± 0.11^c^	5.0 ± 0.5^c^	0.75 ± 0.01^b^	0.15 ± 0.02^cd^
	+ AMF	+ PSF	69.2 ± 4.8^a^	2.23 ± 0.52^b^	7.2 ± 0.2^a^	1.01 ± 0.05^a^	0.14 ± 0.04^d^
170	− AMF	− PSF	0^d^	0^d^	2.8 ± 0.6^f^	0.45 ± 0.01^f^	0.16 ± 0.02^c^
	+ AMF	− PSF	35.3 ± 6.5^c^	0^d^	3.9 ± 0.4^e^	0.70 ± 0.02^d^	0.18 ± 0.04^b^
	− AMF	+ PSF	0^d^	0.75 ± 0.08^c^	4.2 ± 0.5^de^	0.76 ± 0.02^b^	0.18 ± 0.03^b^
	+ AMF	+ PSF	51.1 ± 8.5^b^	4.31 ± 0.59^a^	5.1 ± 0.2^c^	1.07 ± 0.01^a^	0.21 ± 0.03^a^
Salinity (*S*)			**	**	**	*	**
AMF treatment (*A*)			**	**	**	**	ns
PSF treatment (*P*)			**	**	**	**	ns
*S* × *A*			**	**	ns	**	ns
*S* × *P*			ns	**	ns	**	ns
*A* × *P*			**	**	ns	**	ns
*S* × *A* × *P*			ns	**	ns	ns	ns

Data are means ± SE of three replicates. The values of each parameter labeled by different letters indicate significant differences assessed by Duncan’s test after performing a three-way MANOVA (*p* < 0.05). ns non-significant; *significant at *p* < 0.05; **significant at *p* < 0.01.

The impact of salinity was more pronounced in shoots (dry weight reduced by 40%) than roots (dry weight reduced by 20%), which resulted in increasing root/shoot dry weight ratios in beach plum (Table 1). The application of AMF and PSF separately or in combination improved shoot and root growth affected by salt. The dual application of AMF and PSF obviously mitigated the effects of salt stress on all growth parameters, significantly for root growth (dry weight increased by 91.1% compared to non-NaCl; *p* < 0.01).

### Changes in nutrient content of beach plum under salt stress

**Figure 1:**
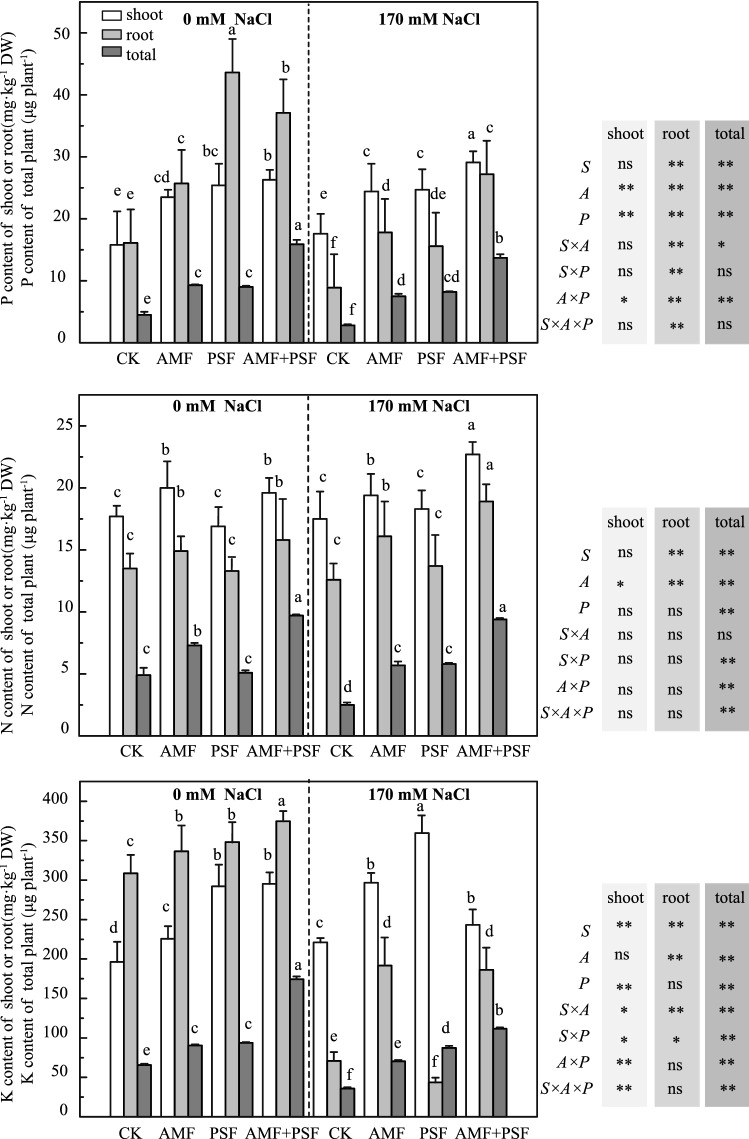
Effects of *F. mosseae* and/or *A. spartima* on the N, P, and K contents of beach plum under saline and non-saline conditions. The values of each parameter labeled by different letters indicate significant differences assessed by Duncan’s test after performing a three-way MANOVA (*p* < 0.05). ns non-significant; *significant at *p* < 0.05; **significant at *p* < 0.01.

Salinity had an adverse effect on P contents in the roots of beach plum but had no significant effect on the shoots (Fig. 1). More P was distributed to roots compared to shoots in non-NaCl-treated plants in the same inoculation treatment, while the reverse was observed in NaCl-treated plants (Fig. 1). However, single and combined inoculation with the AMF and PSF strongly enhanced P contents in both roots and shoots (*p* < 0.01). AMF + PSF treatment exhibited the greatest effects on P contents in the roots and shoots, as well as on the total P contents in plants at 170 mM NaCl.

Compared to non-inoculated plants, inoculation with AMF, PSF, or both significantly improved N concentrations regardless of NaCl stress (Fig. 1, *p* < 0.01). N accumulation was higher in shoots than in roots of the same inoculation treatment (Fig. 1, *p* < 0.01). Among NaCl-treated plants, co-inoculated plants showed the highest N concentrations in the roots and shoots, and the total N content also supported this result. Salinity exhibited no significant effects on N contents in the shoots or roots of non-inoculated or singly inoculated plants (*p* > 0.05).

NaCl stress reduced K contents in the roots but stimulated accumulation in the shoots compared to unstressed plants (Fig. 1). Inoculation with AMF, PSF, or both significantly promoted K accumulation in the roots and shoots regardless of salt stress (*p* < 0.05). Among NaCl-treated plants, PSF-treated plants showed the highest K concentration in the shoots (359.7 mg·kg^−1^ dry weight (DW)) and the lowest values in the roots (43.6 mg·kg^−1^ DW), and co-inoculated plants showed the highest total K concentrations (111.6 mg·kg^−1^ DW).

### Changes in photosynthetic parameters of beach plum under salt stress

**Figure 2:**
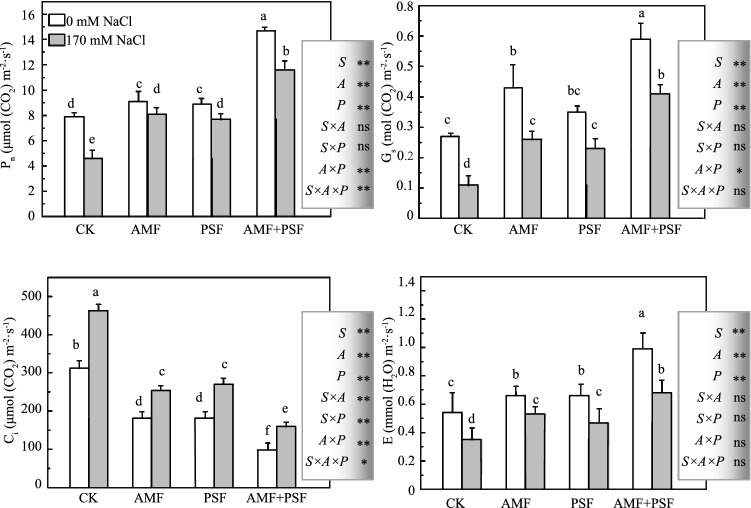
Effects of *F. mosseae* and/or *A. spartima* on the photosynthetic parameters of beach plum under saline and non-saline conditions. Net photosynthetic rate (*P*_n_), intercellular CO_2_ concentration (*C*_i_), stomatal conductance (*G*_*s*_), and transpiration rate (*E*). The values of each parameter labeled by different letters indicate significant differences assessed by Duncan’s test after performing a three-way MANOVA (*p* < 0.05). ns non-significant; *significant at *p* < 0.05; **significant at *p* < 0.01.

The net photosynthetic rate (*P*_n_), stomatal conductance (*G*_*s*_), and transpiration rate (*E*) were higher in inoculated plants than in non-inoculated ones at 0 mM NaCl, while a lower intercellular CO_2_ concentration (*C*_i_) value was observed in inoculated plants. Salinization caused significant (*p* < 0.05) reductions in *P*_n_, *G*_*s*_, and *E* values and a lower *C*_i_ value, while inoculation with AMF, PSF, or both significantly abated such reductions (*p* < 0.05, Fig. 2). The most effective treatment was inoculation with AMF and PSF, and the AMF inoculation treatment yielded *P*_n,_ *G*_*s*,_ *C*_*i*,_ and *E* values close to the PSF inoculation treatment (*p* < 0.05, Fig. 2). Among NaCl-treated plants, plants co-inoculated with AMF and PSF had the highest *P*_n_, *G*_*s*_, and *E* values (increased by 152.1%, 272.7%, and 94.3% compared to only NaCl stress, respectively) and the lowest *C*_*i*_ value (decreased by 65.5% compared to only NaCl stress).

### Changes in Chl content, F_v_/F_m_, ФPSII, qP, and NPQ of beach plum under salt stress

**Figure 3:**
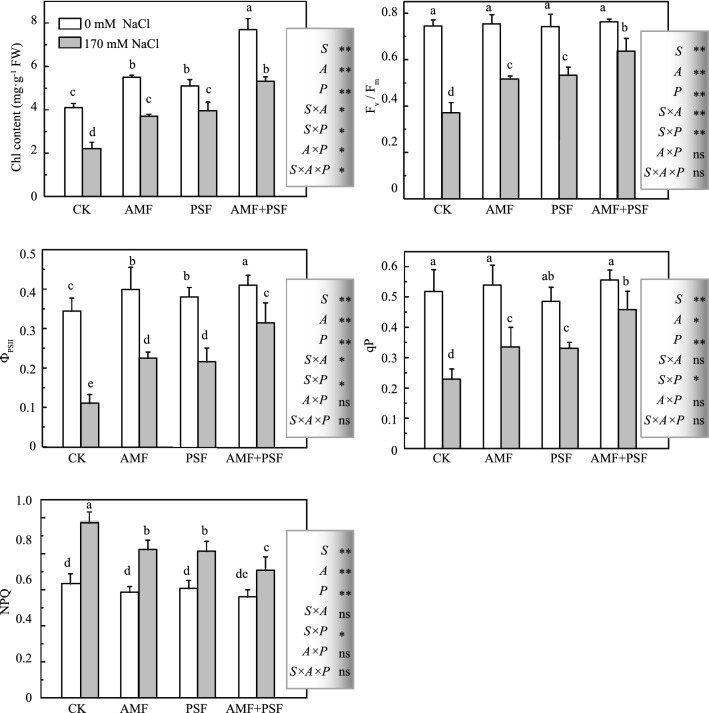
Effect of *F. mosseae* and/or *A. spartima* on the chlorophyll fluorescence parameters of beach plum under saline and non-saline conditions*.* The values of each parameter labeled by different letters indicate significant differences assessed by Duncan’s test after performing a three-way MANOVA (*p* < 0.05). ns non-significant; *significant at *p* < 0.05; **significant at *p* < 0.01.

The maximum efficiency of PSII (F_v_/F_m_), photochemical quenching coefficient (qP), and nonphotochemical quenching (NPQ) values were affected little by inoculation with AMF, PSF, or both under non-NaCl treatments (*p* < 0.05, Fig. 3). In contrast, single and combined inoculation with the AMF and PSF strongly enhanced the Chl content (increased by 24.3⁠–87.8% compared to the control) and actual quantum yield of PSII photochemistry (Ф_PSII_) value. Salinization caused a significant decrease in Chl content, F_v_/F_m_, qP, and Ф_PSII_ values and an increase in NPQ value, while inoculation with AMF, PSF, or both significantly abated such reductions (*p* < 0.05, Fig. 3). Compared with the NaCl treatment alone, plants co-inoculated with AMF and PSF had the highest F_v_/F_m_, qP, and Ф_PSII_ values (increased by 72.5%, 102.2%, and 188.1%, respectively) and lowest NPQ value.

### Principal component analysis

**Figure 4:**
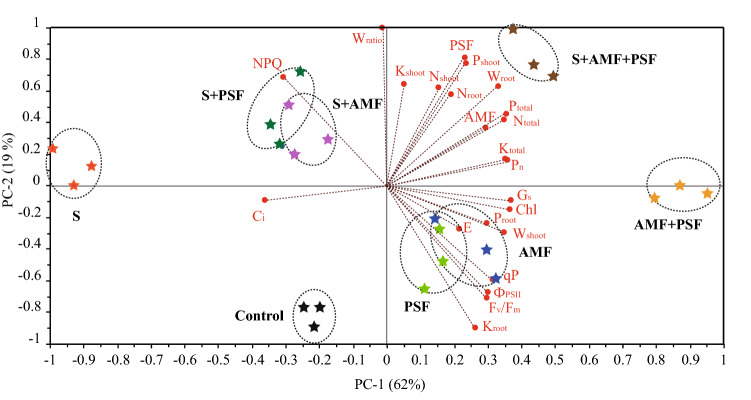
Principal component analysis of the studied parameters of different treatments of beach plum under saline and non-saline conditions. S, Salinity; AMF, arbuscular mycorrhizal fungi; PSF, phosphate-solubilizing fungi; W_shoot_, shoot dry weight; W_root_, root dry weight; W_ratio_, root/shoot dry weight ratio; P_shoot_, phosphorous uptake of shoots; P_root_, phosphorous uptake of roots; P_total_, phosphorous uptake of the total plant; N_shoot_, nitrogen uptake of shoots; N_root_, nitrogen uptake of roots; N_total_, nitrogen uptake of the total plant; K_shoot_, potassium uptake of shoots; K_root_, potassium uptake of roots; K_total_, potassium uptake of the total plant; Chl, chlorophyll; *C*_i_, intercellular CO_2_ concentration; *E,* transpiration rate; F_v_/F_m_, the maximal quantum yield of PSII in dark-adapted state; *G*_*s*_, stomatal conductance; NPQ, nonphotochemical quenching; *P*_n_, net photosynthetic rate; qP, photochemical quenching coefficient; and Ф_PSII_, effective quantum yield of PSII.

Principal component analysis (PCA) accounted for all the parameters of the different treatments. Two main components accounted for 81% of the variability observed in the data, with 62% for PC1 and 19% for PC2 (Fig. 4). Inoculation with AMF or PSF had similar effects regardless of NaCl stress, but the combination of the two fungi was more effective. Under the non-saline condition, PCA indicated there were positive correlations among the growth, fungal infection, nutrient (N, P, and K), Chl content, photosynthetic parameters, and Chl fluorescence parameters but not for C_i_, NPQ, or root/shoot dry weight ratio. PCA also confirmed the negative impact of salt concentration on these parameters, but the combination of AMF and PSF could mitigate these effects, especially for nutrient uptake, root growth, and *P*_n_ (Fig. 4).

## Discussion

### Salinization adversely affected colonization by AMF but positively increased PSF populations

Our results showed that salinity reduced the ability of *F. mosseae* to colonize beach plum roots. This finding was consistent with previous reports that concluded that salinity hinds the spore germination, colonization ability, and the growth of AMF hyphae^18,19^. Our results showed that AMF combined with PSF promoted mycorrhizal root colonization under NaCl stress (Table 1). Combined inoculation with AMF and PSF increased the AMF colonization rate. This may be largely attributed to the increase of soil-soluble P caused by PSF in saline soil, which promoted the rapid colonization of mycorrhiza and increased the distribution, length, and survival rate of the external fungal mycelium in saline soil^20^. The inoculation of AMF and PSF also significantly increased the soil PSF population, possibly due to the carbonaceous substances released by mycorrhized plants, which served as a C source for PSF in the rhizosphere soil^21^.

### AMF and PSF mitigated the effects of salt stress on nutrient uptake, significantly for roots

Co-inoculation with AMF and PSF had a synergistic effect on the increase in plant DW. Similar effects have been found in *Leucaena leucocephala*^22^ and *Kostelelzkya virginica*^4^. *A. spartima* was isolated from the top 0–10 cm of soil from a community of *Spartina alterniflora* in North Jiangsu Province and has been proven to be an efficient phosphate-solubilizing fungus^11^. The contribution of the PSF to growth promotion was probably due to increasing the soil P pool available for AM fungal extraradical hyphae to pass it on to the plants^23^ (Fig. 1). In this study, one reason for the increase in co-inoculated plant DW was thought to be the absorbance of more P from the soil and its accumulation toward the shoots, resulting in increased shoot and root dry weight (Table 1; Fig. 1).

AMF and PSF have a synergistic effect on the dissolution and absorption of P ^10,11,24^. PSF increases the soil P pool that can be used by AMF extraradical hyphae to deliver P to plants^25^. AMF produces extrametrical mycelia, which reduce the distance between P and AMF and allow plants to take up P more easily^16^. However, in the 170 mM NaCl treatment, more P was allocated to the shoots than that to roots in the same inoculation treatment (Fig. 1). The inhibitory effects of salt stress on shoot growth were greater than those on root growth (Table 1), which is consistent with Qiu et al.^11^ and Ben-Laouane et al.^26^ but not with Ait-El-Mokhtar et al.^17^ Salt stress had different effects on the root and shoot growth of different sweet sorghum varieties^10^. Is the varying sensitivity of the roots and stems of different plants to stress related to their particular mechanisms of coping with stress? This question will require us to explore more anatomical and physiological indicators in the future.

Salinity interferes with the acquisition and utilization of N, leading to a decline in plant growth^27^. However, N content in the shoots was not significantly influenced by 170 mM NaCl, while N content in the roots showed an upward trend in the present study (Fig. 1). This may represent an important mechanism for this halophyte to relieve the symptoms of severe salt stress. Regardless of NaCl stress, single inoculation of AMF and co-inoculation with PSF can greatly increase N accumulation in the roots and shoots of beach plum (Table 1; Fig. 1). AMF can increase the utilization of different forms of N by plants^28^, and it has been proven that AMF directly absorbs N and transfers it to the host root system^29^. In addition, AMF and PSF may promote urease activity in the soil, thus catalyzing urea hydrolysis to release ammonia or ammonium ions^18^.

When salt content in the soil is at a high level, plants tend to absorb more Na, resulting in reduced K absorption^30^. Single inoculation with AMF and co-inoculation with PSF could, as Abdel-Fattah and Asrar^31^ also pointed out, significantly promote K accumulation in the roots and shoots compared with non-inoculated plants (Fig. 1). This may be because AM roots explored a larger volume of soil though the extraradical mycelium, which enhanced root exudation or changed rhizosphere pH, thereby increasing K utilization^32^. However, PSF-treated plants had the lowest K concentration in their roots under NaCl stress (Fig. 1). The reason for this is unclear, but the mass propagation of PSF may compete with the root system for K absorption.

### Plants inoculated with AMF and/or PSF showed a positive effect on photosynthesis and Chl fluorescence parameters

Higher chlorophyll content, *P*_n_, *G*_*s*_, and *E* values, and lower *C*_*i*_ values were detected in plants inoculated with AMF, PSF, or both regardless of NaCl stress. This agreed with the results of other studies^11,33^ (Fig. 2). Mycorrhizal inoculation enhances phosphorus and magnesium uptake and reduces sodium concentrations in the plant; this in turn increases Chl content and improves the overall performance of mycorrhizal plants^5^. Van den Driessche^34^ detected a close relationship between some nutrients (N, Mg, and Fe) and chlorophyll and reported that an increase in those nutrients could lead to a stimulation of the synthesis of chlorophyll and hence the photosynthetic capacity. The improvement of gas-exchange parameters in the inoculated plants has been linked to alterations of host plants with the enhanced nutrient uptake ^6,35,36^ and would normally translate into increased photosynthesis^37^. K plays an important role in stomatal movements and protein synthesis^38^. In this study, the increase of K contents in co-inoculated plants is attributed to the accumulation of more K in shoots, resulting in increased *g*_*s*_ values (Figs. 1, 2). These four positive effects, *P*_n_, *G*_*s*_, *C*_*i*_, and *E*, may also have accounted for the enhanced plant growth of colonized plants, most probably by enhancing CO_2_ fixation under salt stress^39^. These also demonstrated that one of the factors resulting in higher dry mass accumulation in inoculated plants, especially in co-inoculated plants, was the increased capacity for CO_2_ assimilation and the decreased intercellular CO_2_ concentration compared to those of non-inoculated plants under NaCl sress^40^.

The parameters of Chl fluorescence accurately reflect photosynthetic ability and energy conversion efficiency^7^. Under salt stress, a reduction in values of F_v_/F_m_, qP, and Ф_PSII_ is often an indicator of photoinhibition or another kind of injury to PSII components^41–43^. Inoculations with AMF increased the salt tolerance of beach plum by reducing the decline in F_v_/F_m_, ΦPSII, and qP and the increase in NPQ caused by NaCl stress (Fig. 3). This indicated that an AMF symbiotic relationship can enhance the efficiency of excitation energy capture by chloroplasts and increase the photochemical capacity of PSII in light-adapted leaves^44^. Co-inoculation with AMF and PSF had a synergistic effect on improvement in the parameters of Chl fluorescence under NaCl stress (Fig. 3). Our data indicated that plants co-inoculated with AMF and PSF had the highest photochemical efficiency for CO_2_ fixation and solar energy utilization and that co-inoculation would significantly reduce the light-induced damage due to salinity compared to a single inoculation with AMF or PSF (Fig. 3). Kaschuk et al.^45^ reported that AM symbiosis enhancing PSII and reducing NPQ may be related to the carbon sink stimulation of AMF symbiosis. Zhang et al.^10^ found that castor bean co-inoculated with AMF and PSF enlarged the C pool of coastal saline soil much more than a single inoculation of AMF or PSF. No information is available on the effects of PSF on Chl fluorescence parameters under NaCl stress to date. PSF inoculation may alleviate the deleterious effects of salt stress on Chl fluorescence parameters by enabling greater nutrient (N, P, and K) absorption^10^ (Fig. 1). Certainly, the hypothesis that inoculations with PSF could improve plant Chl fluorescence parameters under NaCl stress also needs further study. However, among non-NaCl-treated plants, F_v_/F_m_, qP, and NPQ values were not significantly different in the inoculated plants compared to non-inoculated ones (Fig. 3). These parameters are sensitive to stress^6^, and the plants were not subjected to adverse conditions under the condition of no stress.

## Conclusion

Salinity stress reduced growth, nutrient (N, P, and K) uptake, and photosynthetic and chlorophyll fluorescence parameters in beach plum. The application of AMF and PSF separately increased growth parameters, photosynthetic efficiency, and the concentration of photosynthetic pigments under saline conditions by enhancing N, P, and K uptake. These effects could be strengthened by the synergy of AMF and PSF, especially for nutrient uptake, root growth, and *P*_n_. Our findings highlight the importance of considering the dual application of AMF and PSF to alleviate the deleterious effects of beach plum growing in salinized soils.

## Material and methods

### Plant materials

A total of 3000 new, healthy, and semi-lignified branches of beach plum (*Prunus maritima*), each 10-cm-long and 0.5–1.0 cm in diameter with two buds, were collected from the Agricultural Sightseeing Garden, Lishui County, Jiangsu, in March 2015. All cuttings were dipped in 1% (w/w) captan (Red Sun Group, Nanjing, China) for 10 min as a preventative measure against mildew. The substrate used was a 1:1 (v/v) mixture of quartz sand and nutrient soil (a commercial soil purchased from the Red Sun Group, Nanjing, China), which had been sterilized by autoclaving twice for 1 h at 121 °C. The nutrient soil had the following characteristics: pH, 7.05; electric conductivity (EC), 0.71 dS·m^−1^; organic matter, 13.5 g·kg^−1^; hydrolyzable N, 48.0 mg·kg^−1^; available P, 14.7 mg·kg^−1^; and available K, 14.8 g·kg^−1^.

### Fungal inoculum

The mycorrhizal fungus used was *F. mosseae*, mixed in the sandy soils with AMF spores, hyphae, and colonized maize root fragments. The original inoculum (BGCJX01) was provided by the Institute of Plant Nutrition and Fertilizers, Chinese Academy of Agriculture. It was separated from the rhizosphere (neutral to slightly alkaline) of scented *Osmanthus fragrans* trees in Jiangxi, China, and was propagated on maize plants growing in sandy soil for 10 weeks^18^. The *A. spartima* was isolated from the topsoil (0–10 cm) samples of a *Spartina alterniflora* community in North Jiangsu province. It had been previously identified as a phosphate-solubilizing fungus that could significantly enhance available P concentrations^13^. The inoculum of *A. spartima* was prepared using the method of Zhang et al.^4^ To prepare the liquid inoculum of *A. spartima*, the first step was to activate strains on agar slants. The fungus was inoculated on solid Martin culture medium (di-potassium hydrogen phosphate 1 g, magnesium sulfate 0.5 g, sodium chloride 11.5 g, peptone 5 g, glucose 10 g, gelose 10 g, 1/30,000 bengal red water solution 100 mL, and demineralized water 900 mL), which had been autoclaved for 30 min at 121 °C and then incubated in the dark at 28 °C for 4 d. After activation, 3 mL sterile water was added to the test tube, and the mixture was poured into 50 mL Martin broth (MB), which was then added to 1.15% NaCl; *A. spartima* was grown on a rotating shaker at 180 rpm for 48 h, and this was the starter culture. It was added (5% of volume) to MB. We then added 1.15% NaCl, and the MB was cultured on a shaker for 96 h at 180 rpm. At the end, it contained 2.3 × 10^5^ colony forming units per mL, and the solution was stored at 4 °C until use.

### Experimental design and biological treatments

The experiment was run based on a three-factor, two-level randomized block design. One factor, salinity treatment, contained two intensities: 0 and 170 mM NaCl. The second factor, AMF inoculation, *Funneliformis mosseae*, contained two levels: no AMF application (− AMF) and inoculation with AMF (+ AMF). The third factor, PSF inoculation, *Apophysomyces spartima*, included two levels: no PSF application (− PSF) and inoculation with PSF (+ PSF). In total, there were eight treatment combinations, and each was repeated three times. Each replication consisted of 50 pots with one plant per pot, making 1200 pots in total.

On 10 June 2015, 1200 healthy beach plums plants of similar size (height from 49 to 51 cm, number of leaves from 54 to 56) were transplanted into 30 cm × 20 cm pots, each filled with 900 g of cultivation substrate (as above). Referring to the mixing ratio of inocula and substrate in our previous experiments^18^, the fungal inocula were added to the above substrate at a depth of 5 cm with the following design: 10 g *F. mosseae* inoculum and 10 mL sterile *A. spartima* inoculum for the AMF treatment; 10 g sterile *F. mosseae* inoculum and 10 mL *A. spartima* inoculum for the PSF treatment; 10 g *F. mosseae* inoculum and 10 mL *A. spartima* inoculum for the AMF + PSF treatment; and 10 g sterile *F. mosseae* inoculum and 10 mL sterile *A. spartima* inoculum for the control treatment (recorded as CK).

Each pot was placed on a 2-cm-deep tray and placed in a greenhouse under controlled conditions (16 h photoperiod at a light intensity of 220 µmol·m^–2^·s^–1^ at 28 °C, and 8 h of darkness at 18 °C, with the relative humidity kept at 65–85%) on 12 June 2015. All pots were irrigated with distilled water, and the plants were allowed to establish for 4 weeks. The pots were then watered using a modified Hoagland and Arnon^45^ solution with all nutrients except P. The salinity of the substrate was based on the dry mass of the medium and was tested as follows: 3 g of NaCl was dissolved in 300 mL of water and poured into each pot evenly three times between day 7 and day 21 to avoid serious osmotic shock and to give a final NaCl concentration of 170 mM. An equal volume of distilled water was poured into the four trays without NaCl treatment by the same method mentioned above. Any occasional leakage was poured back into the tray after 1 h, and distilled water was added as necessary to maintain the soil moisture level. The pH of the substrate in all treatments was 7.2. After establishing salt stress, each pot was watered with 200 mL of Hoagland and Arnon nutrient solution every 3 d until day 90.

### Plant biomass and nutrient (N, P, K) measurements

After 90 d, three plants were removed randomly from pots and dried in a forced-air oven at 80 °C for 72 h for biomass determination. The oven-dried samples were sieved through a 0.5-mm sieve. A known mass of the ground material was digested in a digestion flask containing a triple acid mixture [HNO_3_:H_2_SO_4_:HCl (60%), with a ratio of 10:1:4] to analyze the total P. P was analyzed using the vanadate-molybdate colorimetric method^10^. Total N was measured in samples of 0.1 g dry mass using the Kjeldahl method^46^. Each sample was heated in a digestion tube for at least 8 h with concentrated sulfuric acid (98.8%). Distillation of the completely digested samples was carried out using an aqueous solution of sodium hydroxide (40%). The extracted ammonium was dissolved in 15 mL of boric acid and then automatically titrated with 25 mmol·L^–1^ sulfuric acid using bromocresol green-methyl red mixture as an indicator. K concentration was analyzed using an ICP (J-A1100; Jarrell-Ash Inc.).

### PSF populations and AMF colonization assessment

Whole plants were extracted from pots on the 30th day after the beginning of the stress period. Soil samples obtained by gentle shaking of the roots were collected in sterilized culture dishes and stored at 4 °C until the PSF population analyses using a tenfold serial dilution test tube technique^47^. To assess the extent of AMF colonization, the roots of three plants from each treatment were cleaned with 10% (w/v) KOH and stained with 0.05% (w/v) trypan blue. The percentage of root length stained and colonized by AMF was estimated according to McGonigle et al.^48^. The roots of each plant were cut into 1-cm-long pieces, and 30 pieces from each plant were examined for their AMF content using a compound microscope (NLCD-307, Ningbo Yongxin Phenix Optical Ltd., Ningbo, China) at 100× magnification. A positive result for AMF colonization included the presence of vesicles or arbuscules, or the typical mycelium within the roots. The percentage of AMF colonization was calculated as follows:

$$ \text{AMF colonization} (\%)  = \text{Colonized Root segments} / \text{Total root segments} \times 100\%. $$

### Gas-exchange parameters measurements

Gas-exchange parameters, including the net photosynthetic rate (*P*_n_), intercellular CO_2_ concentration (*C*_i_), stomatal conductance (*G*_*s*_), and transpiration rate (*E*), were measured using a portable open flow gas-exchange system *LI-6400* (*LI-6400*, *LI-COR*, Lincoln, NE, USA) from 08:30 to 11:30 in the morning^49^. The 3rd to 5th leaves from the end of beach plum were used to assay these parameters. The photosynthetically active irradiation was 1200 µmol·m^–2^·s^–1^, the CO_2_ concentration was 400 cm^3^·m^–3^, the leaf temperature was 25 °C, and the airflow rate was 0.5 dm^3^·min^–1^.

### Chl content and Chl fluorescence parameters measurements

The 3rd to 5th leaves from the end of beach plum were used to assay Chl content and Chl fluorescence parameters. The extraction from 50 mg of fresh material was incubated in 5 mL of 80% acetone in the dark at 4 °C. After incubation, the extract was read at 645 nm and 663 nm in a *Uvikon 940* spectrophotometer with a spectral slit width of 1.8 nm. The following parameters were calculated by the classic formula^49^: Chl *a* = 12.7A_663_ – 2.69A_645_, Chl *b* = 22.9A_645_ – 4.68A_663_. Further, the same leaves were used to measure the parameters of Chl fluorescence according to the methodology of Chen et al.^50^. The *MINI-PAM (MINI-PAM, Waltz*, Germany) was used to measure the fluorescence induction curve and the rapid light response curve of beach plum leaves under salt stress, and in each case, four duplicates of measurement were used. The fluorescence induction curve methods were as follows: Firstly, plants were kept in dark for 30 min before measuring. Secondly, the measuring light was opened (wavelength 650 nm, modulation frequency of 0.6 kHz, photosynthetically active radiation (PAR) less than 0.15 μmol·m^–2^·s^–1^, and the modulation frequency automatically switched to 20 kHz when the saturation pulse light or actinic light opened). Finally, F_0_ was measured. F_m_ was measured by the saturation pulse light (continued 0.8 s, PAR greater than 8000 μmol m^–2^ s^–1^). After 40 s, the actinic light was kept (PAR of about 1000 μmol m^–2^ s^–1^) on and the saturation pulses light was opened every 30 s. The steady-state fluorescence parameters (F_t_) and PAR of the light-adapted sample could be measured when the sample achieved stable status, and the maximum fluorescence yield (F_m_') could be measured under saturation pulse light. The calculated parameters were as follows: F_v_/F_m_ = (F_m_ – F_0_)/F_m_, Ф_PSII_ = (F_m_' – F_t_)/F_m_', qP = (F_m_' – F_t_)/(F_m_' – F_0_), NPQ = (F_m_ – F_m_')/F_m_'.

### Statistical analysis

All data were statistically analyzed by three-way multivariate analysis of variance (MANOVA) for the main effects (Salinity, S; AMF inoculation, A; PSF inoculation, P) and their interactions using SPSS 17.0 (IBM Corp., Armonk, NY, USA), and means were separated using Duncan's test at *p* values < 0.05. Principal component analysis (PCA) was performed using Unscrambler v. 10.4 (CAMO, Oslo, Norway).

## Abbreviations

AM: Carbuscular mycorrhizal
AMF: Arbuscular mycorrhizal fungus
PSF: Phosphate-solubilizing fungus
Chl: Chlorophyll
*C*_i_: Intercellular CO_2_ concentration
DW: Dry weight
*E*: Transpiration rate
F_0_: The minimal fluorescence in a dark-adapted state
F_m_: The maximal fluorescence in a dark-adapted state
F_v_: Variable fluorescence
F_t_: Steady-state fluorescence parameters
F_m_': Maximum fluorescence yield
F_v_/F_m_: The maximal quantum yield of PSII in a dark-adapted state
*G*_*s*_: Stomatal conductance
NPQ: Nonphotochemical quenching
PAR: Photosynthetically active radiation
PSII: Photosystem II
*P*_n_: Net photosynthetic rate
qP: Photochemical quenching coefficient
Ф_PSII_: Effective quantum yield of PSII
